# Behavioral recovery profiling of cockroaches stung by the venomous wasp *Ampulex compressa*

**DOI:** 10.1242/jeb.249768

**Published:** 2025-07-28

**Authors:** Sena Borbor, Stefania Nordio, Frederic Libersat

**Affiliations:** Department of Life Sciences and School of Brain Sciences and Cognition, Ben Gurion University, POB 653, Beer Sheva, Israel 8410501

**Keywords:** Parasitoid wasp, Cockroach, Venom, Dopamine, Grooming

## Abstract

The parasitoid wasp *Ampulex compressa* injects venom into the brain of the American cockroach, targeting the central complex, a sensory-motor region. The venom induces grooming, followed by long-lasting hypokinesia. While grooming is attributed to dopamine in the venom, the mechanisms underlying hypokinesia remain unclear. Given the role of dopamine in modulating arousal and locomotion in insects, and our finding of long-term impairment in venom-induced grooming behavior, we hypothesized that the mechanisms behind long-term grooming impairments may provide insight into the mechanisms driving hypokinesia. We analyzed the recovery profile of venom-induced grooming in stung cockroaches and investigated dopamine receptor involvement through D1-like receptor agonist injections into the central complex. Our results reveal a deficient grooming response to a second sting 1 month after the first, and that this change is not caused by a lasting impairment of D1 receptor signaling but rather a complex interaction between venom components and the recovered brain.

## Teaser

**Summary:** Cockroaches show an impaired grooming response to injection of *Ampulex compressa* venom, 1 month following a first sting, which is not caused by a lasting impairment of D1 receptor signaling.

## INTRODUCTION

The parasitoid wasp *Ampulex compressa* uses the American cockroach (*Periplaneta americana*) as a food source for its larvae. Before laying an egg on the exoskeleton of the cockroach, the wasp injects venom directly into the central complex (CX) (Haspel et al., 2003), a sensory-motor region of the cockroach brain (Pfeiffer and Homberg, 2014; Honkanen et al., 2019). Immediately after the sting, the cockroach grooms for approximately 20 min before entering a long-lasting state of hypokinesia (Libersat, 2003). Although hypokinetic cockroaches are not paralyzed, they show short-term impairments in escape responses and a long-lasting reduction in spontaneous walking (Libersat, 2003).

Insects rely on descending interneurons from the brain to modulate the excitability of thoracic motor circuits, which in turn influence behaviors such as spontaneous walking and escape running (Emanuel and Libersat, 2017; Emanuel et al., 2020; Rana et al., 2023). The CX is a key center for integrating sensory information and guiding context-appropriate movements in insects (Emanuel et al., 2020). It either generates or modulates the descending output that targets reflex pathways and central pattern generators (Emanuel et al., 2020; Kaiser and Libersat, 2015; Rana et al., 2023). Inhibition of CX activity via procaine injections results in decreased spontaneous walking in cockroaches (Kaiser and Libersat, 2015).

It was shown previously that venom-induced grooming is mediated by the activation of D1-like dopamine receptors in the CX (Nordio et al., 2022; Weisel-Eichler et al., 1999). In addition to investigating the dopaminergic basis of venom-induced grooming, Nordio et al. (2022) also demonstrated that dopamine signaling is involved in the venom-induced suppression of escape behavior, which is a short-term component of the hypokinetic state. However, the mechanisms underlying long-term reduction in spontaneous movement remain elusive.

Dopamine plays a central role in modulating arousal and locomotion (Draper et al., 2007; Helfrich-Förster, 2018), and disruptions in dopamine signaling have been associated with locomotor deficits in insects (Draper et al., 2007; Riemensperger et al., 2013). In this context, our observation of a long-term deficit in venom-induced grooming (a behavior known to depend on D1 receptor signaling) led us to hypothesize that the mechanism behind persistent venom-induced grooming impairments may offer insight into the source of long-term hypokinesia. If grooming remains desensitized to venom long after envenomation, this could reflect a long-term alteration in dopamine signaling, which potentially underlies long-term hypokinesia.

We first assessed the grooming response to a second sting administered at various time points using behavioral assays. We then explored the possibility of long-term alterations in dopamine signaling within the CX of stung cockroaches by combining behavioral assays with injections of a D1-like dopamine receptor agonist into the CX.

## MATERIALS AND METHODS

### Animals

The cockroaches, *Periplaneta americana* (Linnaeus 1758), were bred in 50×50×70 cm containers under a 12 h day–night cycle at 26°C, with food (cat chow) and water provided *ad libitum*. The wasps, *Ampulex compressa* (Fabricius 1781), were bred in 40×50×50 cm cages under a 12 h day–night cycle at 30°C, 40% humidity and with honey and water provided *ad libitum*. All experiments were performed on age-matched, male cockroaches. For the long-term (1 month) assays, the cockroaches were kept separately in 18×18×10 cm boxes with sugar water provided *ad libitum*.

The experiments performed comply with Principles of Animal Care (NIH publication no. 86-23, revised in 1985) and with the current laws of the State of Israel.

### Behavior

The behavioral assays for spontaneous grooming and venom/drug-induced grooming were performed in an arena with a radius of 30 cm. Before the quantification of spontaneous grooming, the cockroaches were given 5 min to adjust to the arena. Grooming duration was measured with stopwatches for a period of 30 min. Venom-induced grooming was assessed after a first sting and a second sting delivered at 6 h (*n*=18), 24 h (*n*=10) and 1 month (*n*=7) after receiving the first one.

### Injections

The CX injections followed the protocol described by Kaiser and Libersat (2015) and Nordio et al. (2022). Before the injections, the cockroaches were anesthetized by cold exposure and immobilized with clay on a plate. The neck was gently compressed with a pin to reduce the hemolymph flow to the head for the duration of the injection. The brain was accessed through a small flap opened between the ocelli. The D1-like receptor agonist (±)-SKF-38393 hydrochloride [10^−7^ mol l^−1^, 18.4 nl (9.2 nl+9.2 nl); Sigma] was injected into the CX via a glass capillary connected to a Nanoject II (Drummond Scientific) nano-injector. The cuticle flap was closed and sealed with wax. The behavioral tests started immediately after the injection. The SKF-38393 injections were performed 6 h (*n*=7) or 1 month (*n*=7) after a sting.

### Histology

The accuracy of the injection locations was confirmed only during initial injection training with injections of Janus Green tracer (0.5%; Sigma). Janus Green was dissolved in saline as described in Kaiser and Libersat (2015) and Nordio et al. (2022). The brains were fixed with 10% formalin (Sigma) for 4 h at 25°C and afterwards they were embedded in agar (6% in saline) and cut into 60 µm sections with a vibratome (Leica VT1000S).

### Statistical analysis

The analyses were performed in GraphPad Prism 8.4.2 (GraphPad Software, Boston, MA, USA). Mean differences were analyzed with one-way ANOVA followed by Tukey's multiple comparisons test for the venom-induced grooming assays, and with one-way ANOVA followed by Dunnett's multiple comparisons test for the SKF-38393 injections at the 6 h point and for the acetic acid stimulation assay. The response to SKF-38393 injections and baseline measurements was compared with a paired *t*-test. The response to SKF-38393 injections in the stung (recovered) and control group was compared with a *t*-test.

## RESULTS AND DISCUSSION

**Fig. 1.**
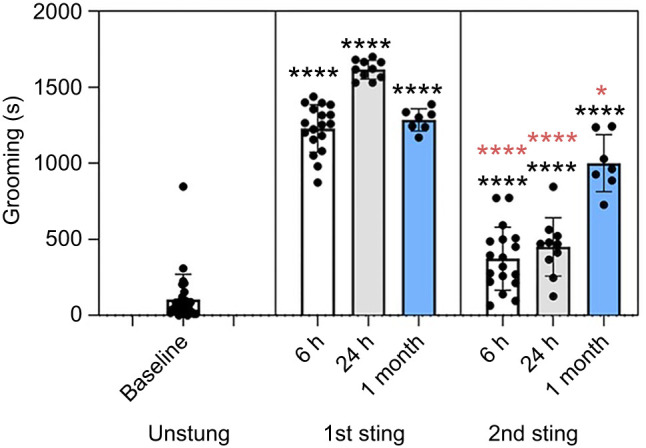
**The grooming response does not fully recover after a month.** Venom-induced grooming after a first sting and a second sting delivered at 6 h (white), 24 h (gray) or 1 month (blue) after the first sting. Black asterisks represent significant differences between the unstung grooming response and the grooming response to a first or a second sting (Tukey's multiple comparisons test, ****P<0.0001, *F*_6,91_=176.7). Red asterisks show significant differences between the first and second stings (Tukey's multiple comparisons test, ****P<0.0001, *P<0.02, *F*_6,91_=176.7). Data are means±s.d.; *n*=18, *n*=10, *n*=7 in 6 h, 24 h and 1 month groups, respectively, for first sting and second sting groups.

Upon envenomation, cockroaches groom intensively for approximately 20 min. Given the involvement of dopamine signaling in mediating venom-induced grooming, we sought to determine whether the venom has long-term effects on the grooming response – potentially reflecting long-term alterations in dopamine signaling. Because hypokinesia is also a long-lasting outcome of envenomation, we hypothesized that persistent grooming deficits could help reveal the mechanisms underlying long-term hypokinesia. To see whether the venom has a long-term effect on the grooming response, the cockroaches were subjected to a second sting at intervals of 6 h, 24 h and 1 month after the first sting, and the grooming duration was measured. Cockroaches stung a second time showed impaired venom-induced grooming at all time points, with a strong impairment at 6 and 24 h (Fig. 1; Tukey's multiple comparisons test, *P*<0.0001). Notably, the grooming response was still impaired at the 1 month time point (Fig. 1; Tukey's multiple comparisons test, *P*<0.02). These results prompted further investigation into whether long-term changes in dopamine signaling, particularly in the CX, might underlie this deficit.

Importantly, cockroaches injected with the irreversible D1/D2 antagonist EEDQ did not show impaired grooming when sprayed with an irritant (Fig. S1; Dunnett's multiple comparisons test, *P*<000.1). Similarly, stung hypokinetic cockroaches and those injected with the D1/D2 antagonist flupentixol also showed a normal grooming response following application of an irritant spray (Weisel-Eichler et al., 1999; Weisel-Eichler and Libersat, 2002). These findings show that the grooming deficit following the second sting is not due to a general inability to initiate grooming in the hypokinetic state. Additionally, by the 1 month mark, the loss of venom responsiveness cannot be attributed to baseline dopamine receptor turnover rates, as full receptor turnover typically occurs within 48 h (Nordio et al., 2022). We hypothesized that this reduction in venom-induced grooming results from a long-term disruption of dopamine signaling following envenomation, potentially through reduced dopamine receptor availability or changes in downstream signal transduction.

**Fig. 2.**
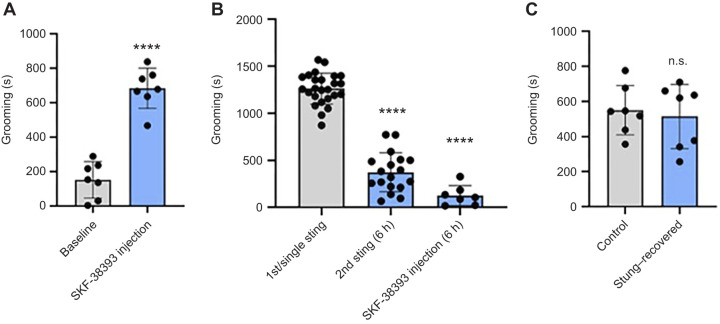
**A second sting or D1 receptor agonist injection into the central complex (CX) 6 h after a sting fails to induce grooming and recovered stung and control (unstung) cockroaches show a similar grooming response following D1 receptor agonist injection received after 1 month.** (A) Grooming response to the injection of the D1 receptor agonist SKF-38393 compared with baseline (paired *t*-test, ****P<0.0001, *n*=7). (B) Grooming response of cockroaches following stings and pharmacological treatment. The first bar represents cockroaches receiving either a single sting or the first of two stings (responses pooled). The second bar shows responses to a second sting delivered 6 h after the first. The third bar represents responses to injection of the dopamine D1 agonist SKF-38393, 6 h after a single sting. Asterisks represent significant differences from the first sting/single sting group (Dunnett's multiple comparisons test, ****P<0.0001, *F*_2,47_=190.2; *n*=25, *n*=18, *n*=7 in 1st/single sting, 2nd sting and SKF-38393 injection groups, respectively). (C) SKF-38393-induced grooming in unstung cockroaches (control) and in stung cockroaches 1 month after a sting (stung–recovered) (*t*-test, *n*=7). There were no significant differences between the groups. Data are means±s.d.

Previous research demonstrated that injecting the D1 receptor agonist SKF-38393 into the CX induces grooming (Nordio et al., 2022). This finding was confirmed by our control experiment (Fig. 2A; paired *t*-test, *P*<0.0001). To investigate whether the venom induces grooming via D1 receptor activation, two groups of stung cockroaches were either injected with SKF-38393 or subjected to a second sting 6 h after the first sting. The cockroaches that received the SKF-38393 injection showed a grooming deficit similar to those that were stung a second time, suggesting a shared mechanism between the venom and the agonist in grooming induction, specifically through D1 receptor signaling (Fig. 2B; Dunnett's multiple comparisons test, *P*<0.0001).

The verification that the venom acts through D1-like dopamine receptors to induce grooming and the persistence of the reduction in venom-induced grooming at the 1 month point led us to investigate a possible long-term effect of the venom on D1 signaling. Hence, stung cockroaches were injected with SKF-38393 1 month after the sting. The grooming response was similar to control levels (Fig. 2C; *t*-test, *P*>0.05). We conclude that the grooming deficit seen in response to a second sting received 1 month after the first does not arise from a long-term impairment in the D1-like dopamine receptor signaling pathway after a first sting. Rather, it may be a result of the interaction of the wasp venom, rich in various components (Arvidson et al., 2019), with the recovered cockroach brain. Future research could address the differential protein expression in the CX of stung–recovered and unstung cockroaches to identify the molecular pathways involved in long-term behavioral alterations.

## Supplementary Material

10.1242/jexbio.249768_sup1 Supplementary information
