# Supplementary material for: Behavioral recovery profiling of cockroaches stung by the venomous wasp Ampulex compressa
Source: J Exp Biol. 2025 Jul 28;228(15):jeb249768. doi: 10.1242/jeb.249768 (PMC12377806; doi:10.1242/jeb.249768)
Supplement: Supplementary information [file jexbio-228-249768-s1.pdf]

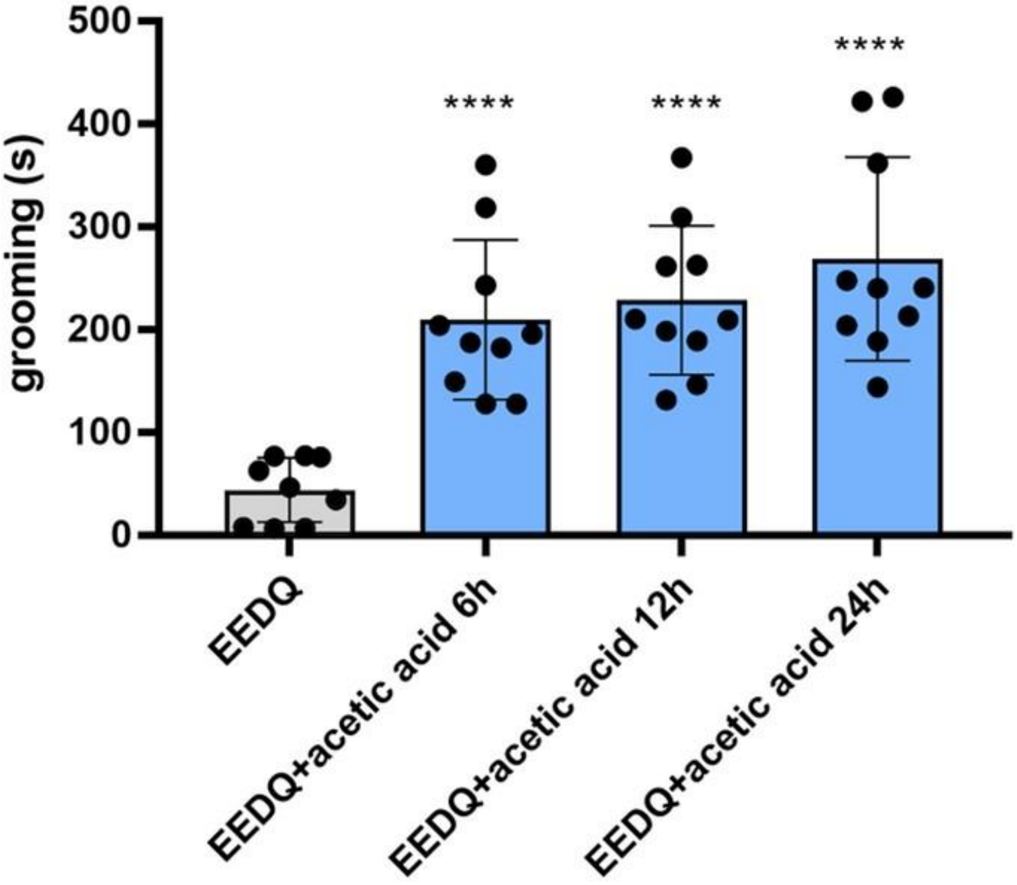

Fig. S1. EEDQ-injected cockroaches show intense grooming behavior following acetic acid stimulation. Spontaneous grooming behavior of EEDQ injected cockroaches (gray) compared to the grooming behavior of EEDQ injected cockroaches sprayed with acetic acid 6, 12 and 24 hours after the EEDQ injection (blue). Asterisks show significant differences between the EEDQ injection group and the EEDQ + acetic acid groups (Dunnett's multiple comparisons test, ****= $p < 0.0001$, $F (3, 35) = 16.12$). n=9, n=10, n=10, n=10 in EEDQ, EEDQ + acetic acid 6h, 12h and 24h groups respectively.

Journal of Experimental Biology • Supplementary information
